# The roles of the mtDNA-cGAS-STING axis in tumor immunity: from immune activation to immune evasion

**DOI:** 10.3389/fimmu.2025.1739559

**Published:** 2026-01-12

**Authors:** Nan Huang, Zheng Liu, Haibo Lei, Xiang Liu

**Affiliations:** Department of Clinical Pharmacy, Xiangtan Central Hospital (The Affiliated Hospital of Hunan University), Xiangtan, China

**Keywords:** immune activation, immune evasion, mtDNA-cGAS-STING, tumor immune microenvironment, tumor therapy

## Abstract

In the tumor microenvironment (TME), stress-induced mitochondrial DNA (mtDNA) leakage activates the mtDNA-cyclic GMP-AMP synthase (cGAS)- stimulator of interferon genes (STING) axis, which exerts a “double-edged sword” role in tumor immunity. On the one hand, it activates the STING- interferon regulatory factor 3 (IRF3) pathway via cyclic GMP-AMP (cGAMP) synthesis by cGAS, induces type I interferons (IFN-I), enhances the cytotoxic functions of CD8^+^ T cells and natural killer (NK) cells as well as the antigen-presenting capacity of dendritic cells (DCs), and also promotes M1 macrophage polarization and neutrophil extracellular trap (NETs) formation, thereby driving immune activation. On the other hand, sustained activation of this axis can induce programmed cell death ligand 1 (PD-L1) expression, recruit myeloid-derived suppressor cells (MDSCs), and cause T cells exhaustion, facilitating tumor immune evasion. Targeting mtDNA stability, constructing nano-drug delivery systems, or combining with immune checkpoint blockade can reshape the tumor immune microenvironment and provide new ideas for precision immunotherapy. This article systematically summarizes the dual effects of this axis on the tumor immune microenvironment, which not only deepens the understanding of cancer immunology but also provides guidance for the research, development, and optimization of precision tumor immunotherapies, and is expected to improve patient prognosis.

## 1 Introduction

The TME is a dynamic ecosystem surrounding tumor cells, comprising immune cells (such as TAMs and regulatory T cells), stromal cells, vascular systems, and cytokines. It plays a central role in tumor initiation, progression, and immune evasion (1–3). As a complex ecosystem, the TME relies on DNA-sensing mechanisms to act as a key link between cellular stress and immune responses (4). mtDNA, a unique immunostimulant, leaks into the cytoplasm from damaged mitochondria when cells are subjected to genomic instability, metabolic stress, or therapy-induced damage (5). This cytoplasmic mtDNA can be recognized by cGAS, which in turn activates the STING-IRF3 signaling pathway, ultimately triggering an inflammatory response (6). Notably, this DNA-sensing mechanism not only participates in antitumor immune responses but is also closely associated with tumor immune evasion (5).

As a circular double stranded DNA (dsDNA) molecule, mtDNA is effectively recognized by DNA sensors due to its structural characteristics and plays a particularly important role in activating the cGAS-STING pathway (7). It is worth noting that the cGAS-STING pathway activated by mtDNA plays a dual role in tumor immunity. On the one hand, this pathway exerts anti-tumor effects by activating host immunity (8, 9); On the other hand, certain tumors may use this pathway to evade immune surveillance (5, 10, 11).

This article focuses on the core scientific question of the dual role of mtDNA-cGAS-STING axis in tumor immunity. We discussed the potential mechanisms by which this pathway participates in immune activation and immune escape, and summarized the drugs currently under research targeting this pathway.

## 2 Mechanisms of mtDNA release and cGAS-STING pathway activation

### 2.1 Pathways of mtDNA release induced by mitochondrial stress

mtDNA is typically sequestered within the mitochondrial matrix and enclosed by mitochondrial nucleoids (12). Under stress conditions, however, it can be released from damaged mitochondria into the cytoplasm and extracellular space, serving as a key immunostimulatory signal. This release is triggered by various stressors, including oxidative stress, viral infection, and drug toxicity (9), that activate distinct but often overlapping mechanisms. These mechanisms can be classified into four major pathways based on the underlying cellular process: (i) Membrane permeabilization pathways represent primary routes for mtDNA release. Mitochondrial outer membrane permeabilization (MOMP), mediated by BAX/BAK-dependent membrane depolarization (13, 14) and promoted by PGAM5-mediated Bax translocation (15), is prominently induced by severe oxidative stress, viral infection, and apoptosis-inducing drugs. Alternatively, mitochondrial inner membrane permeability transition (MIMP) can independently mediate mtDNA release through progressive widening of outer membrane pores coupled with increased inner membrane permeability, a process particularly associated with calcium overload and metabolic toxicity (13, 16). (ii) Vesicular transport provides a membrane-preserving mechanism for mtDNA export. Mitochondrial-derived vesicles (MDVs) selectively transport mtDNA to the cytoplasm, serving as an alternative release pathway activated by metabolic stress conditions (e.g., fumarate accumulation) and during viral infection (16). (iii) Quality control failure leads to catastrophic mtDNA release. Defective mitophagy causes accumulation of damaged mitochondria, resulting in spontaneous mtDNA release through organelle rupture under conditions of prolonged stress, aging, and drug toxicity (17). (iv) Direct molecular destabilization of the mtDNA-nucleoid complex facilitates leakage. Downregulation of mitochondrial transcription factor A (TFAM), triggered by nutrient stress and viral infection, impairs mtDNA stability and promotes its release (7, 18). Overexpression of Drp1 induces mitochondrial dysfunction and stress-induced leakage, particularly under bioenergetic stress conditions (19). Additionally, excessive oxygen species (ROS) production, a hallmark of oxidative stress, oxidizes mtDNA and directly facilitates its translocation into the cytoplasm and extracellular environment (20, 21).

### 2.2 Involvement of mtDNA in the activation of the cGAS-STING pathway

The cGAS- STING pathway is a core component of the innate immune system that specifically recognizes cytoplasmic dsDNA to initiate host immune responses (22). This pathway plays a crucial role in tumor immunity, antiviral responses, and autoimmune diseases (23). mtDNA in the cytosol is specifically recognized by cGAS, an enzyme that possesses unique structural features as a dsDNA sensor (24). cGAS forms a complex with negatively charged mtDNA via its positively charged DNA-binding domain; this binding induces a conformational change in cGAS and exposes its catalytic pocket (25). Experimental evidence has shown that either digestion of mtDNA by DNase I or reduction of cytosolic mtDNA using ethidium bromide (EtBr) can significantly inhibit cGAS activation (7, 26, 27). Notably, oxidatively modified mtDNA exhibits stronger cGAS-binding capacity, suggesting that oxidative stress may amplify this signaling pathway (21, 28).

Upon activation, cGAS produces the second messenger cyclic GMP-AMP (cGAMP), which binds to endoplasmic reticulum (ER)-localized STING protein and induces its conformational change (29). Activated STING translocates to the Golgi apparatus region, where it recruits and phosphorylates TANK-binding kinase 1 (TBK1) (25). Subsequently, TBK1 phosphorylates the transcription factor IRF3, promoting the dimerization of IRF3 and its translocation to the nucleus (30, 31). This cascade reaction can be affected by multiple regulatory mechanisms: epigenetic silencing of STING blocks signal transduction (5), while overexpression of mitochondrial TFAM reduces STING activation by stabilizing mtDNA (32, 33). Super-resolution imaging technology has confirmed a clear spatiotemporal correlation between mtDNA release and STING activation (13).

After translocating into the nucleus, activated IRF3 initiates the transcription of type I interferon (IFN-α/β) genes (25). These interferons activate the JAK-STAT pathway through autocrine and paracrine effects, inducing the expression of hundreds of interferon-stimulated genes (ISGs) (5, 34, 35). Overall, upon binding cytosolic mtDNA, cGAS synthesizes the second messenger 2’3’-cGAMP, activating STING on the ER membrane. STING then recruits TBK1 and IRF3, triggering IRF3 phosphorylation and nuclear translocation to drive IFN-I transcription. IFN-I is a pleiotropic cytokine with antiviral, antiproliferative, and immunomodulatory functions. In tumors, IFN-I can activate anti-tumor immune responses by recruiting natural killer cells and expanding CD4+ and CD8+ T cells; conversely, mild and persistent IFN-I signaling can lead to immunosuppression by inducing mediators involved in T cell exhaustion, such as PD-L1, IDO, and IL-10 (36). Studies have shown that STING-deficient mice exhibit a significantly attenuated inflammatory response (37, 38), whereas the introduction of exogenous mtDNA can restore IFN-β production (39, 40). Notably, this pathway can also synergistically activate the NLRP3 inflammasome, facilitating the release of pro-inflammatory cytokines such as IL-1β (21, 41).

## 3 mtDNA-cGAS-STING axis promotes tumor immune activation (**Figure 1**)

### 3.1 Mechanisms of NK cell and T cells activation

NK cells are cytotoxic lymphocytes with the ability to kill tumor cells and secrete pro-inflammatory cytokines (42). Owing to their role in tumor suppression, NK cells play a critical function in tumor immune surveillance, particularly in preventing tumor metastasis (43). The mtDNA-cGAS-STING pathway activates cytotoxic immune cells through multiple mechanisms. In NK cells, mtDNA released by tumor cells partially triggers intrinsic STING activation in NK cells via recognition by cGAS, thereby maintaining the antitumor activity of the TCF-1+ NK cell subset (a subset with long-term memory potential) (8).

T cells exhibit multiple mechanisms of action in tumor therapy and provide robust support for tumor treatment through various approaches, including directly killing tumor cells, regulating immune responses, enhancing immune memory, and participating in immune checkpoint inhibition (44, 45). In particular, CD8+ T cells can directly recognize and kill tumor cells: they identify complexes of tumor-specific antigens (TSAs) or tumor-associated antigens (TAAs) with MHC class I molecules on the surface of tumor cells via T cell receptors (TCRs). Once recognition occurs, cytotoxic T lymphocytes (CTLs) release cytotoxic granules, such as perforin and granzyme, which can induce tumor cell apoptosis (46). In CD8+ T cells, IFN-I produced after STING pathway activation can promote their proliferation and the expression of cytotoxic granules (47). Experimental evidence shows that direct activation of STING using cyclic GMP-AMP (cGAMP) significantly enhances the IFN-γ production capacity of NK cells; meanwhile, the ferroptosis-induced mtDNA-releasing can effectively recruit CD8+ T cells infiltration into tumors by activating the STING pathway (48). Additionally, the cGAS-STING axis activated by cytoplasmic mtDNA can promote the occurrence of pyroptosis, which in turn activates CD8+ T cells in a paracrine manner (49).

### 3.2 Maturation of DCs and enhancement of antigen presentation

DCs play a crucial role as a link between innate and adaptive immunity in the immune system and serve as important hubs for immune responses. As a bridge connecting innate and adaptive immunity, DCs have attracted significant attention due to their excellent antigen-presenting ability (50). In the process of antitumor immunity, DCs perform an indispensable and vital function; their roles are closely associated with the cancer-immune cycle, ultimately facilitating the elimination of tumor cells by effector T cells (51). Studies have revealed that DCs are key effector cells for the activation of the cGAS-STING pathway (52, 53). Acute STING activation promotes DCs function through two mechanisms: first, it directly induces the upregulated expression of DCs maturation markers (e.g., CD80, CD86); second, it enhances DCs’ antigen-presenting ability, which mainly depends on the autocrine loop of IFN-I triggered by mtDNA (47, 54, 55). Immunogenic cell death (ICD) induced by photothermal therapy can synergize with STING pathway activation to promote the uptake and processing of tumor antigens by DCs, forming a positive feedback loop (55).

### 3.3 Neutrophil extracellular traps and TME

NETs are mesh-like structures released by neutrophils, mainly composed of DNA, histones, and related proteases. The release of NETs is usually triggered by reactive ROS, which subsequently activates peptidylarginine deiminase 4 (PAD4), leading to the citrullination of histone 3 (H3) and further causing DNA unwinding and nuclear membrane rupture (56). Immediately after, gasdermin D (GSDMD)-mediated cell perforation further results in the release of DNA, histones, and related proteases, a process termed NETosis (57). During the release of NETs, mtDNA leakage often occurs, and the interaction between mtDNA and the cGAS-STING signaling pathway can sometimes promote the release of NETs (24, 58). In addition, various cell types can capture NETs through phagocytosis by recipient cells; intracellular cGAS recognizes NETs-derived DNA, thereby activating the cGAS-STING signaling pathway and increasing the expression levels of interleukin-6 (IL-6) and IFN-I (59). Most myeloid cells, such as macrophages and DCs, have been reported to recognize NETs-DNA intracellularly (60). Other epithelial-derived cells and tumor cells can also take up NETs and recognize them via intracellular cGAS (61). For example, hepatic tumor cells possess the ability to take up NETs, thereby activating the cGAS-STING pathway and inhibiting tumor migration (62). In summary, the release of NETs can amplify inflammation mediated by the mtDNA-cGAS-STING pathway, while activation of the mtDNA-cGAS-STING pathway can in turn promote the release of NETs—yet the specific mechanisms remain unclear.

### 3.4 Activation of macrophages

Tumor-associated macrophages (TAMs) are among the most abundant immune cells in the TME. Their plasticity allows them to differentiate into antitumor M1-like phenotypes or protumor M2-like phenotypes. Classically activated M1 macrophages possess potent tumor-killing, phagocytic, and antigen-presenting capacities; however, TAMs in the TME are typically educated into M2-like phenotypes, which assist tumor progression by supporting angiogenesis, promoting tumor proliferation, and establishing an immunosuppressive network (63–65). Therefore, targeting and reprogramming the activation status of TAMs has emerged as a crucial strategy to enhance antitumor efficacy. Recent studies have shown that cytoplasmic mtDNA induces inflammatory cascades by activating the cGAS-STING-IRF3 signaling axis, effectively reshaping macrophage function—this not only inhibits their protumor activities but also significantly enhances their antigen-presenting capacity (66, 67). For instance, when exogenous mtDNA is taken up by TAMs, it can directly drive their conversion from a protumor to an antitumor phenotype, providing a novel intervention target for cancer immunotherapy (68).

## 4 The mtDNA-cGAS-STING axis involved in tumor immune evasion (**Figure 1**)

### 4.1 Immunosuppressive polarization of TAMs

The mtDNA-cGAS-STING axis exerts dual roles in regulating the polarization of TAMs. In the hepatocellular carcinoma model, mtDNA induces the polarization of TAMs toward the M2 subtype through the TLR9- nuclear factor κB (NF-κB) signaling, forming an immunosuppressive microenvironment (69, 70); however, whether the cGAS-STING signaling is involved remains to be further studied. There is evidence that cGAS-STING activation induced by mtDNA can activate JAK–STAT3 signaling (71), and this signaling plays an important role in the differentiation of TAMs into the M2 subtype (72–74). Interestingly, oxidatively modified mtDNA can escape from tumor cells and act as an immunogenic damage-associated molecular pattern to induce the polarization of TAMs toward the M1 subtype, thereby reactivating the immune response of macrophages against cancer cells (75, 76). These findings reveal the complex role of mtDNA-cGAS-STING axis in regulating macrophage polarization in shaping the TME.

**Figure 1:**
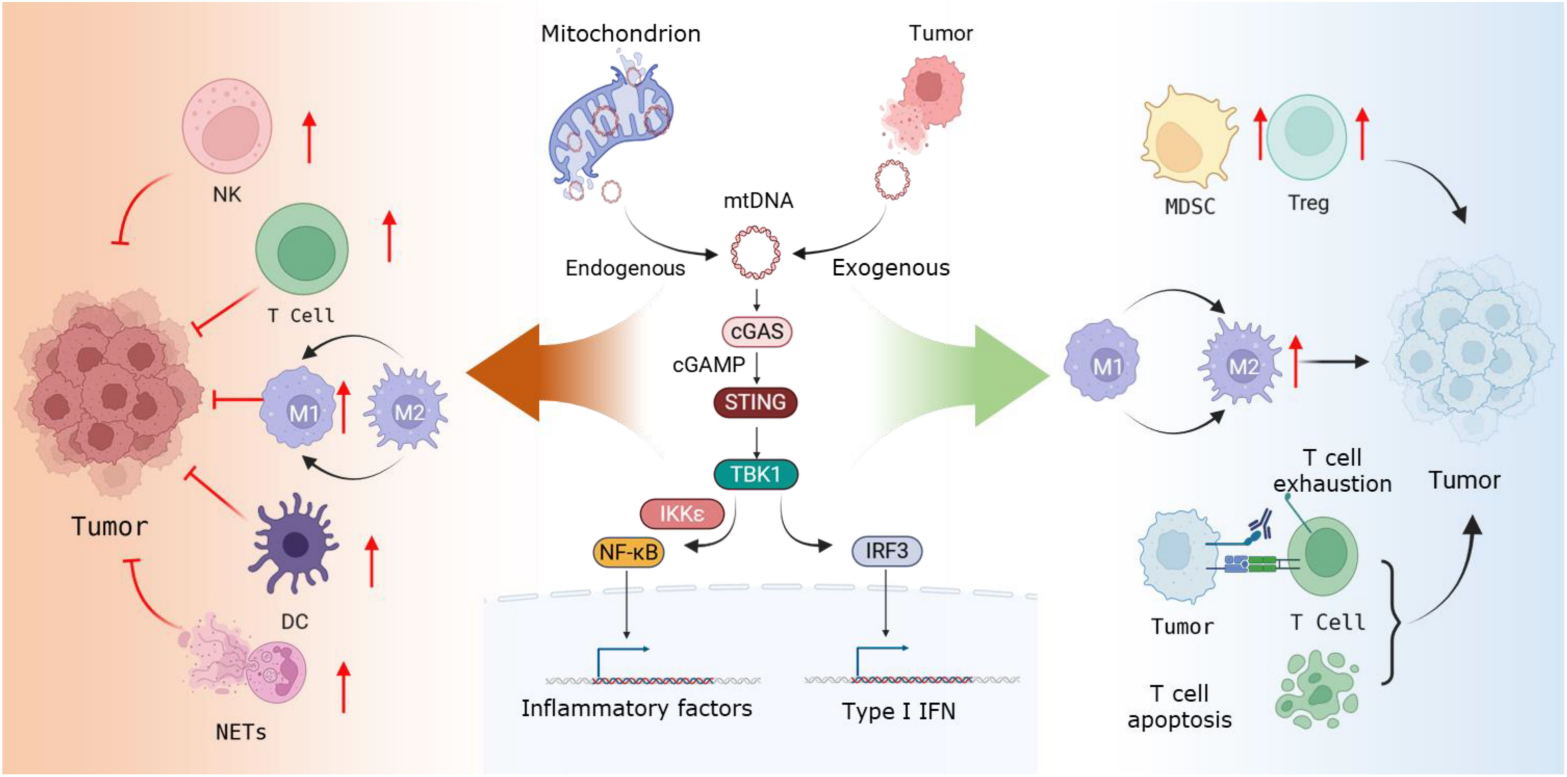
Mechanism diagram of mtDNA-cGAS-STING Axis involvement in tumor immune regulation. Endogenous or exogenous mtDNA activates the cGAS-STING axis. On the one hand, it exerts immunostimulatory effects and inhibits tumor growth by activating NK cells, T cells, DCs, and NETs, as well as promoting macrophage differentiation toward the M1 subtype; On the other hand, it facilitates immune evasion and supports tumor cell growth and metastasis by enhancing the immunosuppressive activity of MDSCs and Treg cells, promoting macrophage differentiation toward the M2 subtype, and inducing T cell exhaustion and apoptosis.

### 4.2 Coordinated regulation of the mtDNA-cGAS-STING axis and PD-1/PD-L1 pathway in tumor immune evasion

The mtDNA-cGAS-STING axis critically regulates tumor immune evasion through coordinated interaction with the PD-1/PD-L1 pathway. Activation of this axis promotes IFN-I secretion, which upregulates PD-L1/PD-1 expression and drives T cell exhaustion (77–79). Vesicle-mediated mechanisms further integrate these pathways. Necrotic tumor cells release extracellular vesicles (EVs) enriched in mtDNA and PD-L1 that induce macrophage IFN/IL-6 production to weaken T cell responses while directly triggering T cell apoptosis (80, 81). Similarly, IL-6-induced EVs promote mtDNA leakage in endometrial cancer, an effect reversible by anti-PD-L1 therapy (80). Although the precise role of cGAS-STING activation in these vesicle-mediated processes requires further investigation, these findings establish a strong rationale for combination therapies targeting both the mtDNA-cGAS-STING pathway and immune checkpoints.

### 4.3 mtDNA-cGAS-STING axis and MDSCs in tumor immune escape

The core function of MDSCs is to construct an immunosuppressive microenvironment in tumors, which inhibits anti-tumor immune responses through various mechanisms, while directly or indirectly promoting tumor growth, metastasis, and drug resistance (82). mtDNA released by senescent cells can be packaged in extracellular vesicles, which are selectively transferred to polymorphonuclear myeloid-derived suppressor cells (PMN-MDSCs) in TEM. This process enhances the immunosuppressive activity of PMN-MDSCs via the cGAS-STING-NF-κB signaling (83). Additionally, exogenous mtDNA can also activate the STING pathway, which in turn creates an immunosuppressive microenvironment in MDSCs. This ultimately provides favorable conditions for the survival and proliferation of tumor cells (83, 84).

## 5 The potential mechanism of double sided immunoregulation of mtDNA-cGAS-STING axis

Acute and chronic activation of the mtDNA-cGAS-STING signaling pathway exhibit distinct differences in their underlying mechanisms and biological effects, with core disparities manifested in the duration of activation, intensity of signal transduction, and the ultimate outcomes of the mediated immune responses (77). Nevertheless, these two activation modes share a common feature: both can effectively activate IRF3 and NF-κB signaling pathways, thereby inducing dual effects of immune activation and immunosuppression in the organism (85–87).

Mitochondrial damage induced by short-term chemotherapy, or acute oxidative stress triggers massive acute release of mtDNA into the cytoplasm. The leaked mtDNA potently activates the cGAS-STING pathway, which in turn induces the phosphorylation of TBK1 (5). Phosphorylated TBK1 further activates IRF3 and NF-κB signaling, leading to the secretion of large amounts of type I interferons (IFN-α/β) and inflammatory factors (31). This immune activation effect can enhance antitumor immune responses, specifically characterized by strengthened T cell-mediated tumor cell killing and promoted maturation of DCs as well as their antigen-presenting function (77).

In the context of chronic oxidative stress, sustained elevation of reactive ROS results in persistent mtDNA leakage, which also activates the cGAS-STING pathway but with significantly lower signal intensity compared to acute activation. The low-intensity and sustained signal stimulation ultimately contributes to the formation of an immunosuppressive microenvironment and promotes tumor progression (77). Typical biological effects include sustained high expression of PD-L1, recruitment of MDSCs with enhanced immunosuppressive activity, accumulation of regulatory T cells (Tregs), and impairment of effector T cell infiltration (88, 89).

These observations suggest that the combination of radiotherapy/chemotherapy with STING agonists may exert a synergistic antitumor effect, but chronic activation of the pathway should be strictly avoided. In clinical practice, pulsatile stimulation is superior to sustained stimulation as an administration strategy, as it can enhance immune activation while minimizing the risk of immunosuppression.

Notably, the STING signaling pathway can activate NF-κB through a redundant mechanism involving TBK1 and IκB kinase ϵ (IKKϵ) (90). This mechanistic characteristic determines that a single TBK1 inhibitor cannot fully block NF-κB activation but can completely abrogate IRF3 signaling. This finding implies that specific signal blockers may serve as molecular “switches” for the precise regulation of IRF3 and NF-κB pathways, providing a novel direction for the optimization of immunotherapeutic strategies.

## 6 Clinical trials targeting the mtDNA-cGAS-STING axis

Clinical trials related to STING agonists and ENPP1 inhibitors in tumors are showing a pattern of active exploration and gradual advancement. According to data from ClinicalTrials.gov (as of December 1, 2025), 15 oncology-related studies have been registered for STING agonists, mostly in Phase I; research on ENPP1 inhibitors in tumors is still in the early exploratory stage, with a total of 4 studies all in Phase I, among which 3 are actively recruiting participants and 1 has not yet initiated recruitment (**Table 1**). Currently, no clinical data on safety and efficacy have been accumulated for ENPP1 inhibitors. Among STING agonist studies, most were terminated due to adjustments in corporate business policies, while TAK-500 and MIW815 were discontinued due to the lack of observed definite antitumor activity, reflecting the complexity of clinical development for this class of drugs.

**Table 1:** Summary of clinical trials targeting the mtDNA-cGAS-STING axis.

Class	Drug	NCT number	Phase	Status	Year
STING agonists	CRD3874	NCT06626633	Phase 1	Active, not recruiting	2024-2028
STING agonists	E7766	NCT04109092	Phase 1	Withdrawn	2020-2022
STING agonists	E7766	NCT04144140	Phase 1	Terminated	2020-2022
STING agonists	CRD3874	NCT06021626	Phase 1	Recruiting	2023-2029
STING agonists	IMSA101	NCT06601296	Phase 2	Recruiting	2025-2028
STING agonists	IMSA101	NCT05846659	Phase 2	Terminated	2023-2024
STING agonists	IMSA101	NCT05846646	Phase 2	Terminated	2023-2024
STING agonists	TAK-500	NCT05070247	Phase 1 Phase 2	Terminated	2022-2025
STING agonists	MIW815	NCT03172936	Phase 1	Terminated	2017-2020
STING agonists	MIW815	NCT02675439	Phase 1	Terminated	2016-2020
STING agonists	MIW815	NCT03937141	Phase 2	Terminated	2019-2021
STING agonists	SNX281	NCT04609579	Phase 1	Terminated	2020-2024
STING agonists	MK-1454	NCT03010176	Phase 1	Completed	2017-2022
STING agonists	MK-1454	NCT04220866	Phase 2	Completed	2020-2022
STING agonists	PF-07820435	NCT06285097	Phase 1	Terminated	2024-2025
ENPP1 inhibitors	SR-8541A	NCT06063681	Phase 1	Recruiting	2023-2025
ENPP1 inhibitors	TXN10128	NCT05978492	Phase 1	Recruiting	2023-2026
ENPP1 inhibitors	ISM5939	NCT06724042	Phase 1	Not yet recruiting	2025-2029
ENPP1 inhibitors	RBS2418	NCT05270213	Phase 1	Recruiting	2022-2027

Indicates that there is no combination drug.

Notably, the STING agonist MK-1454 has preliminarily validated its antitumor potential and safety through two clinical studies: Phase I (NCT03010176) and Phase II (NCT04220866) (91). In terms of antitumor activity, MK-1454 has demonstrated clear target-binding ability and synergistic therapeutic effects: in the Phase I study (N = 156), plasma drug concentrations increased in a dose-dependent manner, and key STING pathway-related cytokines in the circulation, such as CXCL10, IFNγ, and IL-6, began to increase 2–4 hours after administration, peaked at 6–8 hours, and partially declined by 24 hours, directly confirming that the drug can effectively activate the STING pathway; the exploratory combination therapy for head and neck squamous cell carcinoma (HNSCC) and triple-negative breast cancer in the expansion phase of this study provided an important direction for the expansion of its clinical application scenarios. In the Phase II randomized controlled study, for treatment-naïve patients with metastatic or unresectable recurrent HNSCC, the objective response rate (ORR) of MK-1454 combined with pembrolizumab reached 50% (4/8), which was significantly higher than the 10% (1/10) of pembrolizumab monotherapy, clearly demonstrating the synergistic antitumor advantages of the combination regimen.

In terms of safety, the toxicity profile of MK-1454 is controllable and manageable. The most common adverse event in the Phase I study was pyrexia (incidence of 70%), and only 10 patients experienced dose-limiting toxicities (DLTs), based on which 540 μg was identified as the recommended Phase II dose; in the Phase II study, pyrexia remained the main adverse event (n=5), with no serious adverse events endangering patients’ safety. Neither monotherapy nor combination therapy with pembrolizumab showed an intolerable toxicity profile. In summary, existing data preliminarily confirm that drugs targeting the mtDNA-cGAS-STING pathway (such as MK-1454) have considerable antitumor potential. However, current studies have limitations such as small sample sizes and short follow-up periods. Efficacy heterogeneity and long-term safety still need to be further clarified through larger-scale and longer-cycle clinical studies. Nevertheless, existing explorations have provided key references for the subsequent clinical translation and optimization of treatment regimens for this class of drugs.

## 7 Conclusion and outlook

The mtDNA–cGAS–STING axis plays a Janus-faced role in tumor immunity: acute engagement ignites type-I interferon signaling that empowers immune cells and antitumor responses, whereas chronic activation sculpts an immunosuppressive niche that enables immune evasion. Its functional output is shaped by tumor subtype, microenvironment milieu, and signal intensity.

While existing studies have achieved considerable progress, several key limitations remain unresolved. First, in terms of mtDNA quantification, existing methods have a technical constraint in differentiating DNA sources (nuclear vs. mitochondrial DNA) during leakage, which may compromise the accuracy of functional interpretations related to mtDNA. Super-resolution imaging, as a cutting-edge tool with high spatial resolution, can effectively visualize the subcellular localization of DNA and distinguish the distribution of nuclear DNA from mitochondrial DNA. The application of this technology in future studies will help address the current limitation and improve the reliability of mtDNA leakage detection. Second, there is the limitation of model systems. Immune-deficient models cannot fully recapitulate STING-dependent immune crosstalk in the physiological tumor microenvironment, as they lack a functional adaptive immune system. Therefore, the findings derived from these models should be interpreted with caution when extrapolated to clinical settings. To enhance the generalizability of existing research findings, we suggest further validating them in humanized mouse models.

To date, most insights derive from *in-vitro* or animal models, with limited clinical corroboration; precise tools to titrate mtDNA release and robust STING-targeted delivery platforms are still missing. Furthermore, cross-talk with other immune-regulatory circuits remains poorly charted, constraining the design of optimal combination regimens. Future efforts must map spatiotemporal control of this axis across human cancers, engineer accurate strategies to modulate mtDNA leakage and STING activity, integrate nanotechnology-based delivery systems with combinatorial immunotherapies, and rigorously validate safety and efficacy, thereby accelerating the clinical translation of personalized cancer immunotherapy.

## References

[1] Kao KC Vilbois S Tsai CH Ho PC . Metabolic communication in the tumor-immune microenvironment. Nat Cell Biol. (2022) 24:1574–83. doi: 10.1038/s41556-022-01002-x, PMID: 36229606

[2] Tang T Huang X Zhang G Hong Z Bai X Liang T . Advantages of targeting the tumor immune microenvironment over blocking immune checkpoint in cancer immunotherapy. Signal transduction targeted Ther. (2021) 6:72. doi: 10.1038/s41392-020-00449-4, PMID: 33608497 PMC7896069

[3] Zhu S Wang Y Tang J Cao M . Radiotherapy induced immunogenic cell death by remodeling tumor immune microenvironment. Front Immunol. (2022) 13:1074477. doi: 10.3389/fimmu.2022.1074477, PMID: 36532071 PMC9753984

[4] Decout A Katz JD Venkatraman S Ablasser A . The cGAS-STING pathway as a therapeutic target in inflammatory diseases. Nat Rev Immunol. (2021) 21:548–69. doi: 10.1038/s41577-021-00524-z, PMID: 33833439 PMC8029610

[5] Aloraini GS . Mitochondrial DNA release and cGAS-STING activation: Emerging insights into anti-tumor immunity. Pathology Res practice. (2025) 273:156158. doi: 10.1016/j.prp.2025.156158, PMID: 40774059

[6] Gu X Chen Y Cao K Tu M Liu W Ju J . Therapeutic landscape in systemic lupus erythematosus: mtDNA activation of the cGAS-STING pathway. Int immunopharmacology. (2024) 133:112114. doi: 10.1016/j.intimp.2024.112114, PMID: 38652968

[7] Yang J Yang M Wang Y Sun J Liu Y Zhang L . STING in tumors: a focus on non-innate immune pathways. Front Cell Dev Biol. (2023) 11:1278461. doi: 10.3389/fcell.2023.1278461, PMID: 37965570 PMC10642211

[8] Lu L Yang C Zhou X Wu L Hong X Li W . STING signaling promotes NK cell antitumor immunity and maintains a reservoir of TCF-1(+) NK cells. Cell Rep. (2023) 42:113108. doi: 10.1016/j.celrep.2023.113108, PMID: 37708030

[9] Chen J Liang S Li C Li B He M Li K . Mitochondrial damage causes inflammation via cGAS-STING signaling in ketamine-induced cystitis. Inflammation Res. (2025) 74:6. doi: 10.1007/s00011-024-01973-7, PMID: 39762437 PMC11703929

[10] Konno H Yamauchi S Berglund A Putney RM Mulé JJ Barber GN . Suppression of STING signaling through epigenetic silencing and missense mutation impedes DNA damage mediated cytokine production. Oncogene. (2018) 37:2037–51. doi: 10.1038/s41388-017-0120-0, PMID: 29367762 PMC6029885

[11] Timilsina S Huang JY Abdelfattah N Medina D Singh D Abdulsahib S . Epigenetic silencing of DNA sensing pathway by FOXM1 blocks stress ligand-dependent antitumor immunity and immune memory. Nat Commun. (2025) 16:3967. doi: 10.1038/s41467-025-59186-3, PMID: 40295473 PMC12037779

[12] Farge G Falkenberg M . Organization of DNA in mammalian mitochondria. Int J Mol Sci. (2019) 20:1–14. doi: 10.3390/ijms20112770, PMID: 31195723 PMC6600607

[13] Riley JS Quarato G Cloix C Lopez J O’Prey J Pearson M . Mitochondrial inner membrane permeabilization enables mtDNA release during apoptosis. EMBO J. (2018) 37:1–16. doi: 10.15252/embj.201899238, PMID: 30049712 PMC6120664

[14] Allen ER Whitefoot-Keliin KM Palmatier EM Mahon AR Greenlee-Wacker MC . Extracellular vesicles from A23187-treated neutrophils cause cGAS-STING-dependent IL-6 production by macrophages. Front Immunol. (2022) 13:949451. doi: 10.3389/fimmu.2022.949451, PMID: 35967325 PMC9374307

[15] Li J Sun X Yang N Ni J Xie H Guo H . Phosphoglycerate mutase 5 initiates inflammation in acute kidney injury by triggering mitochondrial DNA release by dephosphorylating the pro-apoptotic protein Bax. Kidney Int. (2023) 103:115–33. doi: 10.1016/j.kint.2022.08.022, PMID: 36089186

[16] Zecchini V Paupe V Herranz-Montoya I Janssen J Wortel IMN Morris JL . Fumarate induces vesicular release of mtDNA to drive innate immunity. Nature. (2023) 615:499–506. doi: 10.1038/s41586-023-05770-w, PMID: 36890229 PMC10017517

[17] Newman LE Weiser Novak S Rojas GR Tadepalle N Schiavon CR Grotjahn DA . Mitochondrial DNA replication stress triggers a pro-inflammatory endosomal pathway of nucleoid disposal. Nat Cell Biol. (2024) 26:194–206. doi: 10.1038/s41556-023-01343-1, PMID: 38332353 PMC11026068

[18] Li Y Yang Q Chen H Yang X Han J Yao X . and ESCC survival through mtDNA stress-mediated STING pathway. Oncogene. (2022) 41:3735–46. doi: 10.1038/s41388-022-02365-z, PMID: 35750756

[19] Li Y Chen H Yang Q Wan L Zhao J Wu Y . Increased Drp1 promotes autophagy and ESCC progression by mtDNA stress mediated cGAS-STING pathway. J Exp Clin Cancer research: CR. (2022) 41:76. doi: 10.1186/s13046-022-02262-z, PMID: 35209954 PMC8867650

[20] Waseem M Imtiaz A Alexander A Graham L Contreras-Galindo R . Crosstalk between oxidative stress, mitochondrial dysfunction, chromosome instability, and the activation of the cGAS-STING/IFN pathway in systemic sclerosis. Ageing Res Rev. (2025) 110:102812. doi: 10.1016/j.arr.2025.102812, PMID: 40562314

[21] Yang H Sun P Zhou S Tang Y Li S Li W . Chlamydia psittaci infection induces IFN-I and IL-1beta through the cGAS-STING-IRF3/NLRP3 pathway via mitochondrial oxidative stress in human macrophages. Veterinary Microbiol. (2024) 299:110292. doi: 10.1016/j.vetmic.2024.110292, PMID: 39581075

[22] Samson N Ablasser A . The cGAS-STING pathway and cancer. Nat cancer. (2022) 3:1452–63. doi: 10.1038/s43018-022-00468-w, PMID: 36510011

[23] Hopfner KP Hornung V . Molecular mechanisms and cellular functions of cGAS-STING signaling. Nat Rev Mol Cell Biol. (2020) 21:501–21. doi: 10.1038/s41580-020-0244-x, PMID: 32424334

[24] Xia L Yan X Zhang H . Mitochondrial DNA-activated cGAS-STING pathway in cancer: Mechanisms and therapeutic implications. Biochim Biophys Acta Rev cancer. (2025) 1880:189249. doi: 10.1016/j.bbcan.2024.189249, PMID: 39701325

[25] Hu MM Shu HB . Mitochondrial DNA-triggered innate immune response: mechanisms and diseases. Cell Mol Immunol. (2023) 20:1403–12. doi: 10.1038/s41423-023-01086-x, PMID: 37932533 PMC10687031

[26] Ju YN Li H Zhuo ZP Yang Q Gao W . Mitochondrial DNA from endothelial cells activated the cGAS-STING pathway and regulated pyroptosis in lung ischemia reperfusion injury after lung transplantation. Immunobiology. (2025) 230:152865. doi: 10.1016/j.imbio.2024.152865, PMID: 39826223

[27] Hu M Zhou M Bao X Pan D Jiao M Liu X . ATM inhibition enhances cancer immunotherapy by promoting mtDNA leakage and cGAS/STING activation. J Clin Invest. (2021) 131:1–15. doi: 10.1172/jci139333, PMID: 33290271 PMC7843232

[28] Qiao W Hu C Ma J Dong X Dalangood S Li H . Low-dose metronomic chemotherapy triggers oxidized mtDNA sensing inside tumor cells to potentiate CD8(+)T anti-tumor immunity. Cancer letters. (2023) 573:216370. doi: 10.1016/j.canlet.2023.216370, PMID: 37660883

[29] Li T Chen ZJ . The cGAS-cGAMP-STING pathway connects DNA damage to inflammation, senescence, and cancer. J Exp Med. (2018) 215:1287–99. doi: 10.1084/jem.20180139, PMID: 29622565 PMC5940270

[30] Fitzgerald KA McWhirter SM Faia KL Rowe DC Latz E Golenbock DT . IKKepsilon and TBK1 are essential components of the IRF3 signaling pathway. Nat Immunol. (2003) 4:491–6. doi: 10.1038/ni921, PMID: 12692549

[31] Yum S Li M Fang Y Chen ZJ . TBK1 recruitment to STING activates both IRF3 and NF-κB that mediate immune defense against tumors and viral infections. Proc Natl Acad Sci United States America. (2021) 118:1–9. doi: 10.1073/pnas.2100225118, PMID: 33785602 PMC8040795

[32] Zhao M Wang Y Li L Liu S Wang C Yuan Y . Mitochondrial ROS promote mitochondrial dysfunction and inflammation in ischemic acute kidney injury by disrupting TFAM-mediated mtDNA maintenance. Theranostics. (2021) 11:1845–63. doi: 10.7150/thno.50905, PMID: 33408785 PMC7778599

[33] Liu H Zhen C Xie J Luo Z Zeng L Zhao G . TFAM is an autophagy receptor that limits inflammation by binding to cytoplasmic mitochondrial DNA. Nat Cell Biol. (2024) 26:878–91. doi: 10.1038/s41556-024-01419-6, PMID: 38783142

[34] Darnell JE Jr. Kerr IM Stark GR . Jak-STAT pathways and transcriptional activation in response to IFNs and other extracellular signaling proteins. Sci (New York NY). (1994) 264:1415–21. doi: 10.1126/science.8197455, PMID: 8197455

[35] Zanin N Viaris de Lesegno C Podkalicka J Meyer T Gonzalez Troncoso P Bun P . STAM and Hrs interact sequentially with IFN-α Receptor to control spatiotemporal JAK-STAT endosomal activation. Nat Cell Biol. (2023) 25:425–38. doi: 10.1038/s41556-022-01085-6, PMID: 36797476

[36] Vella V De Francesco EM Bonavita E Lappano R Belfiore A . IFN-I signaling in cancer: the connection with dysregulated Insulin/IGF axis. Trends Endocrinol metabolism: TEM. (2022) 33:569–86. doi: 10.1016/j.tem.2022.04.009, PMID: 35691786

[37] Luo X Li H Ma L Zhou J Guo X Woo SL . Expression of STING is increased in liver tissues from patients with NAFLD and promotes macrophage-mediated hepatic inflammation and fibrosis in mice. Gastroenterology. (2018) 155:1971–1984.e4. doi: 10.1053/j.gastro.2018.09.010, PMID: 30213555 PMC6279491

[38] Zhang Q Wei J Liu Z Huang X Sun M Lai W . STING signaling sensing of DRP1-dependent mtDNA release in kupffer cells contributes to lipopolysaccharide-induced liver injury in mice. Redox Biol. (2022) 54:102367. doi: 10.1016/j.redox.2022.102367, PMID: 35724543 PMC9218162

[39] Ouyang W Wang S Yan D Wu J Zhang Y Li W . The cGAS-STING pathway-dependent sensing of mitochondrial DNA mediates ocular surface inflammation. Signal transduction targeted Ther. (2023) 8:371. doi: 10.1038/s41392-023-01624-z, PMID: 37735446 PMC10514335

[40] Li Q Wang S Guo P Feng Y Yu W Zhang H . Mitochondrial DNA release mediated by TFAM deficiency promotes copper-induced mitochondrial innate immune response via cGAS-STING signaling in chicken hepatocytes. Sci total environment. (2023) 905:167315. doi: 10.1016/j.scitotenv.2023.167315, PMID: 37742962

[41] Yang NS Zhong WJ Sha HX Zhang CY Jin L Duan JX . mtDNA-cGAS-STING axis-dependent NLRP3 inflammasome activation contributes to postoperative cognitive dysfunction induced by sevoflurane in mice. Int J Biol Sci. (2024) 20:1927–46. doi: 10.7150/ijbs.91543, PMID: 38481801 PMC10929193

[42] Morvan MG Lanier LL . NK cells and cancer: you can teach innate cells new tricks. Nat Rev Cancer. (2016) 16:7–19. doi: 10.1038/nrc.2015.5, PMID: 26694935

[43] López-Soto A Gonzalez S Smyth MJ Galluzzi L . Control of metastasis by NK cells. Cancer Cell. (2017) 32:135–54. doi: 10.1016/j.ccell.2017.06.009, PMID: 28810142

[44] Chow A Perica K Klebanoff CA Wolchok JD . Clinical implications of T cell exhaustion for cancer immunotherapy. Nat Rev Clin Oncol. (2022) 19:775–90. doi: 10.1038/s41571-022-00689-z, PMID: 36216928 PMC10984554

[45] Zebley CC Zehn D Gottschalk S Chi H . T cell dysfunction and therapeutic intervention in cancer. Nat Immunol. (2024) 8):1344–54. doi: 10.1038/s41590-024-01896-9, PMID: 39025962 PMC11616736

[46] Philip M Schietinger A . CD8(+) T cell differentiation and dysfunction in cancer. Nat Rev Immunol. (2022) 22:209–23. doi: 10.1038/s41577-021-00574-3, PMID: 34253904 PMC9792152

[47] Wu Y Lu WM Cui QR Zhou J Lu GD . Metabolic regulation of cGAS-STING signaling in the tumor microenvironment: dual immune roles and therapeutic implications. Cytokine Growth factor Rev. (2025) 85:43–55. doi: 10.1016/j.cytogfr.2025.06.002, PMID: 40517100

[48] Liang JL Jin XK Zhang SM Huang QX Ji P Deng XC . Specific activation of cGAS-STING pathway by nanotherapeutics-mediated ferroptosis evoked endogenous signaling for boosting systemic tumor immunotherapy. Sci bulletin. (2023) 68:622–36. doi: 10.1016/j.scib.2023.02.027, PMID: 36914548

[49] Xu X Lu X Zheng Y Xie Y Lai W . Cytosolic mtDNA-cGAS-STING axis mediates melanocytes pyroptosis to promote CD8(+) T-cell activation in vitiligo. J Dermatol science. (2025) 117:61–70. doi: 10.1016/j.jdermsci.2024.12.002, PMID: 39904676

[50] Sarkar SK Willson AML Jordan MA . The plasticity of immune cell response complicates dissecting the underlying pathology of multiple sclerosis. J Immunol Res. (2024) 2024:5383099. doi: 10.1155/2024/5383099, PMID: 38213874 PMC10783990

[51] Marciscano AE Anandasabapathy N . The role of dendritic cells in cancer and anti-tumor immunity. Semin Immunol. (2021) 52:101481. doi: 10.1016/j.smim.2021.101481, PMID: 34023170 PMC8545750

[52] Ribeiro ARS Neuper T Horejs-Hoeck J . The role of STING-mediated activation of dendritic cells in cancer immunotherapy. Int J nanomedicine. (2024) 19:10685–97. doi: 10.2147/ijn.S477320, PMID: 39464674 PMC11512692

[53] Li X Dong Y Wang T Huang K Guo W Xu L . Chemotherapy boosts anti-angiogenic and anti-PD-1 combination therapy through activation of cCAS-STING pathway in colon cancer. Int immunopharmacology. (2025) 149:114212. doi: 10.1016/j.intimp.2025.114212, PMID: 39904029

[54] Li G Zhao X Zheng Z Zhang H Wu Y Shen Y . cGAS-STING pathway mediates activation of dendritic cell sensing of immunogenic tumors. Cell Mol Life sciences: CMLS. (2024) 81:149. doi: 10.1007/s00018-024-05191-6, PMID: 38512518 PMC10957617

[55] Pan X Lin Y Lin C Liu S Lin P Lin X . Enhanced cGAS-STING activation and immune response by LPDAM platform-based lapachone-chemical-photothermal synergistic therapy for colorectal cancer. Advanced healthcare materials. (2025) 14:e2403309. doi: 10.1002/adhm.202403309, PMID: 40103499

[56] Li P Li M Lindberg MR Kennett MJ Xiong N Wang Y . PAD4 is essential for antibacterial innate immunity mediated by neutrophil extracellular traps. J Exp Med. (2010) 207:1853–62. doi: 10.1084/jem.20100239, PMID: 20733033 PMC2931169

[57] Fuchs TA Abed U Goosmann C Hurwitz R Schulze I Wahn V . Novel cell death program leads to neutrophil extracellular traps. J Cell Biol. (2007) 176:231–41. doi: 10.1083/jcb.200606027, PMID: 17210947 PMC2063942

[58] Messaoud-Nacer Y Culerier E Rose S Maillet I Rouxel N Briault S . STING agonist diABZI induces PANoptosis and DNA mediated acute respiratory distress syndrome (ARDS). Cell Death disease. (2022) 13:269. doi: 10.1038/s41419-022-04664-5, PMID: 35338116 PMC8953969

[59] Apel F Andreeva L Knackstedt LS Streeck R Frese CK Goosmann C . The cytosolic DNA sensor cGAS recognizes neutrophil extracellular traps. Sci Signaling. (2021) 14:1–16. doi: 10.1126/scisignal.aax7942, PMID: 33688080

[60] Cao Y Shi M Liu L Zuo Y Jia H Min X . Inhibition of neutrophil extracellular trap formation attenuates NLRP1-dependent neuronal pyroptosis via STING/IRE1α pathway after traumatic brain injury in mice. Front Immunol. (2023) 14:1125759. doi: 10.3389/fimmu.2023.1125759, PMID: 37143681 PMC10152368

[61] Chen J Wang T Li X Gao L Wang K Cheng M . DNA of neutrophil extracellular traps promote NF-κB-dependent autoimmunity via cGAS/TLR9 in chronic obstructive pulmonary disease. Signal transduction targeted Ther. (2024) 9:163. doi: 10.1038/s41392-024-01881-6, PMID: 38880789 PMC11180664

[62] Li N Zheng X Chen M Huang L Chen L Huo R . Deficient DNASE1L3 facilitates neutrophil extracellular traps-induced invasion via cyclic GMP-AMP synthase and the non-canonical NF-κB pathway in diabetic hepatocellular carcinoma. Clin Trans Immunol. (2022) 11:e1386. doi: 10.1002/cti2.1386, PMID: 35474906 PMC9021716

[63] Dalton WB Ghiaur G Resar LM . Taking the STING out of acute myeloid leukemia through macrophage-mediated phagocytosis. J Clin Invest. (2022) 132:1–3. doi: 10.1172/jci157434, PMID: 35229728 PMC8884892

[64] Caiazza C Brusco T D’Alessio F D’Agostino M Avagliano A Arcucci A . The lack of STING impairs the MHC-I dependent antigen presentation and JAK/STAT signaling in murine macrophages. Int J Mol Sci. (2022) 23:1–16. doi: 10.3390/ijms232214232, PMID: 36430709 PMC9697192

[65] Tan H Cai M Wang J Yu T Xia H Zhao H . Harnessing macrophages in cancer therapy: from immune modulators to therapeutic targets. Int J Biol Sci. (2025) 21:2235–57. doi: 10.7150/ijbs.106275, PMID: 40083710 PMC11900799

[66] Wang XD Liu YS Chen MD Hu MH . Discovery of a triphenylamine-based ligand that targets mitochondrial DNA G-quadruplexes and activates the cGAS-STING immunomodulatory pathway. Eur J medicinal Chem. (2024) 269:116361. doi: 10.1016/j.ejmech.2024.116361, PMID: 38547736

[67] Liu J Xiang J Jin C Ye L Wang L Gao Y . Medicinal plant-derived mtDNA via nanovesicles induces the cGAS-STING pathway to remold tumor-associated macrophages for tumor regression. J nanobiotechnology. (2023) 21:78. doi: 10.1186/s12951-023-01835-0, PMID: 36879291 PMC9990354

[68] Cheng P Yang Q Zhang X Wang Q Zhong B . Activation of cGAS-STING pathway by DAI-triggered ferroptosis in CRC cells reprograms TAMs balance to promote anti-tumor immunity. Cancer Sci. (2025) 118:3286–99. doi: 10.1111/cas.70196, PMID: 41021777 PMC12666479

[69] Yang Q Cui M Wang J Zhao Y Yin W Liao Z . Circulating mitochondrial DNA promotes M2 polarization of tumor associated macrophages and HCC resistance to sorafenib. Cell Death disease. (2025) 16:153. doi: 10.1038/s41419-025-07473-8, PMID: 40038250 PMC11880550

[70] Wei X Wang H Liu H Wang J Zhou P Li X . Disruption of tumor-intrinsic PGAM5 increases anti-PD-1 efficacy through the CCL2 signaling pathway. J immunotherapy Cancer. (2025) 13:1–19. doi: 10.1136/jitc-2024-009993, PMID: 39773565 PMC11749670

[71] Tu Q Li Y Zhu J Guo L Liu C Liu L . Mitochondrial DNA mediates immunoparalysis of dendritic cells in sepsis via STING signaling. Cell proliferation. (2022) 55:e13328. doi: 10.1111/cpr.13328, PMID: 36106559 PMC9715356

[72] Li Y Li M Zheng J Ma Z Yu T Zhu Y . Ultrasound-responsive nanocarriers delivering siRNA and fe(3)O(4) nanoparticles reprogram macrophages and inhibit M2 polarization for enhanced NSCLC immunotherapy. ACS Appl materials interfaces. (2024) 16:56634–52. doi: 10.1021/acsami.4c10036, PMID: 39378273

[73] Ou Y Jiang HM Wang YJ Shuai QY Cao LX Guo M . The Zeb1-Cxcl1 axis impairs the antitumor immune response by inducing M2 macrophage polarization in breast cancer. Am J Cancer Res. (2024) 14:4378–97. doi: 10.62347/uais7070, PMID: 39417185 PMC11477816

[74] Wu Z Zhou J Chen F Yu J Li H Li Q . 13-Methyl-palmatrubine shows an anti-tumor role in non-small cell lung cancer via shifting M2 to M1 polarization of tumor macrophages. Int immunopharmacology. (2022) 104:108468. doi: 10.1016/j.intimp.2021.108468, PMID: 35066343

[75] Jiang H Guo Y Wei C Hu P Shi J . Nanocatalytic innate immunity activation by mitochondrial DNA oxidative damage for tumor-specific therapy. Advanced materials (Deerfield Beach Fla). (2021) 33:e2008065. doi: 10.1002/adma.202008065, PMID: 33797131

[76] Jiang Q Chen Z Jiang J Chen Q Lan H Zhu J . The role of cGAS-STING in remodeling the tumor immune microenvironment induced by radiotherapy. Crit Rev oncology/hematology. (2025) 209:104658. doi: 10.1016/j.critrevonc.2025.104658, PMID: 39956501

[77] Huang Z Zhu J Zhou YL Shi J . The cGAS-STING pathway: a dual regulator of immune response in cancer and therapeutic implications. J Trans Med. (2025) 23:766. doi: 10.1186/s12967-025-06843-2, PMID: 40640925 PMC12247247

[78] Zhu W Rao J Zhang LH Xue KM Li L Li JJ . OMA1 competitively binds to HSPA9 to promote mitophagy and activate the cGAS-STING pathway to mediate GBM immune escape. J immunotherapy Cancer. (2024) 12:1–19. doi: 10.1136/jitc-2023-008718, PMID: 38604814 PMC11015223

[79] Tufail M Jiang CH Li N . Immune evasion in cancer: mechanisms and cutting-edge therapeutic approaches. Signal transduction targeted Ther. (2025) 10:227. doi: 10.1038/s41392-025-02280-1, PMID: 40739089 PMC12311175

[80] Zeng X Li X Zhang Y Cao C Zhou Q . IL6 Induces mtDNA Leakage to Affect the Immune Escape of Endometrial Carcinoma via cGAS-STING. J Immunol Res. (2022) 2022:3815853. doi: 10.1155/2022/3815853, PMID: 35692503 PMC9184159

[81] Cheng AN Cheng LC Kuo CL Lo YK Chou HY Chen CH . Mitochondrial Lon-induced mtDNA leakage contributes to PD-L1-mediated immunoescape via STING-IFN signaling and extracellular vesicles. J immunotherapy Cancer. (2020) 8:1–15. doi: 10.1136/jitc-2020-001372, PMID: 33268351 PMC7713199

[82] He S Zheng L Qi C . Myeloid-derived suppressor cells (MDSCs) in the tumor microenvironment and their targeting in cancer therapy. Mol cancer. (2025) 24:5. doi: 10.1186/s12943-024-02208-3, PMID: 39780248 PMC11707952

[83] Lai P Liu L Bancaro N Troiani M Cali B Li Y . Mitochondrial DNA released by senescent tumor cells enhances PMN-MDSC-driven immunosuppression through the cGAS-STING pathway. Immunity. (2025) 58:811–25. doi: 10.1016/j.immuni.2025.03.005, PMID: 40203808

[84] Nambiar DK Viswanathan V Cao H Zhang W Guan L Chamoli M . Galectin-1 mediates chronic STING activation in tumors to promote metastasis through MDSC recruitment. Cancer Res. (2023) 83:3205–19. doi: 10.1158/0008-5472.Can-23-0046, PMID: 37409887 PMC10592379

[85] ZhangBC PedersenA ReinertLS LiY NaritaR IdornM . STING signals to NF-κB from late endolysosomal compartments using IRF3 as an adaptor. Nat Immunol. (2025) 26:1916–30. doi: 10.1038/s41590-025-02283-8, PMID: 40973797

[86] Zhou S Cheng F Zhang Y Su T Zhu G . Engineering and delivery of cGAS-STING immunomodulators for the immunotherapy of cancer and autoimmune diseases. Accounts Chem Res. (2023) 56:2933–43. doi: 10.1021/acs.accounts.3c00394, PMID: 37802125 PMC10882213

[87] Neufeldt CJ Cerikan B Cortese M Frankish J Lee JY Plociennikowska A . SARS-CoV-2 infection induces a pro-inflammatory cytokine response through cGAS-STING and NF-κB. Commun Biol. (2022) 5:45. doi: 10.1038/s42003-021-02983-5, PMID: 35022513 PMC8755718

[88] Du SS Chen GW Yang P Chen YX Hu Y Zhao QQ . Radiation Therapy Promotes Hepatocellular Carcinoma Immune Cloaking via PD-L1 Upregulation Induced by cGAS-STING Activation. Int J Radiat oncology biology physics. (2022) 112:1243–55. doi: 10.1016/j.ijrobp.2021.12.162, PMID: 34986380

[89] Leuzzi G Vasciaveo A Taglialatela A Chen X Firestone TM Hickman AR . SMARCAL1 is a dual regulator of innate immune signaling and PD-L1 expression that promotes tumor immune evasion. Cell. (2024) 187:861–81. doi: 10.1016/j.cell.2024.01.008, PMID: 38301646 PMC10980358

[90] Balka KR Louis C Saunders TL Smith AM Calleja DJ D’Silva DB . TBK1 and IKKϵ Act redundantly to mediate STING-induced NF-κB responses in myeloid cells. Cell Rep. (2020) 31:107492. doi: 10.1016/j.celrep.2020.03.056, PMID: 32268090

[91] Harrington KJ Champiat S Brody JD Cho BC Romano E Golan T . Phase I and II clinical studies of the STING agonist ulevostinag with and without pembrolizumab in participants with advanced or metastatic solid tumors or lymphomas. Clin Cancer research: an. (2025) 31:3400–11. doi: 10.1158/1078-0432.Ccr-24-3630, PMID: 40499147

